# Heart rate variability in cardiovascular disease diagnosis, prognosis and management

**DOI:** 10.3389/fcvm.2025.1680783

**Published:** 2026-01-26

**Authors:** Brian Xiangzhi Wang, Ella Brennand, Pierre Le Page, Andrew R. J. Mitchell

**Affiliations:** 1 Department of Medicine, Jersey General Hospital, St. Helier, Jersey; 2 Department of Medicine, John Radcliffe Hospital, Oxford University Hospitals, Oxford, United Kingdom

**Keywords:** cardiovascular disease, heart rate variability, sudden cardiac death, vagus nerve stimulation, wearable device

## Abstract

Heart rate variability (HRV), the variation in intervals between consecutive heartbeats, reflects autonomic nervous system function and has been studied as a potential biomarker in cardiovascular disease (CVD). While reduced HRV has been linked to arrhythmias, heart failure, and ischaemic heart disease, findings across studies are mixed and its prognostic value remains debated. This review evaluates HRV's diagnostic, prognostic, and therapeutic roles in CVD. HRV can reveal autonomic dysfunction early, predict outcomes such as sudden cardiac death and recurrent myocardial infarction, and track recovery after cardiac events. It also shows promise in monitoring comorbid conditions like heart failure and depression that exacerbate cardiovascular risk. Advancements in wearable technology and machine learning are expanding HRV's potential. Wearable devices enable continuous, non-invasive HRV monitoring, while machine learning algorithms enhance the precision and predictive power of HRV analysis. These innovations may facilitate real-time data collection and tailored treatment plans, though their clinical utility requires validation in larger, prospective trials. Key challenges remain, including measurement variability, lack of standardisation, and limited incremental prognostic value over established risk factors. This review highlights HRV's emerging role in personalised cardiovascular care while acknowledging the substantial research needed before widespread clinical adoption.

## Graphical Abstract

**Figure:**
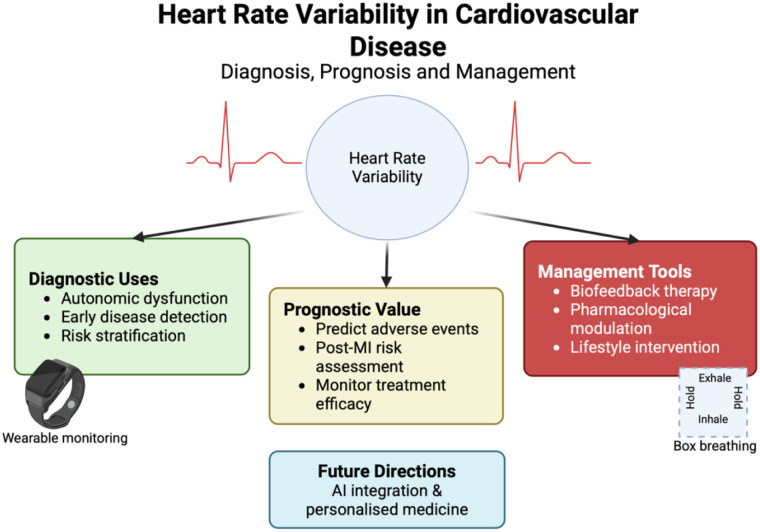
Heart rate variability (HRV) in cardiovascular disease. HRV reflects autonomic regulation of cardiac function and has been studied across three clinical domains: diagnostic applications (autonomic dysfunction, early disease detection, risk stratification), prognostic value (prediction of adverse events, post–myocardial infarction risk, treatment monitoring), and management tools (biofeedback therapy, pharmacological modulation, and lifestyle interventions). Advances in wearable monitoring enable longitudinal HRV assessment, while future directions include artificial intelligence–assisted analysis and personalised medicine. Box breathing is shown as an example of a structured breathing technique used in HRV biofeedback.

## 1 Introduction

Cardiovascular diseases (CVDs) remain the leading cause of morbidity and mortality worldwide, accounting for over 20 million deaths annually (1). Early detection, risk stratification, and effective management are critical in reducing its health and economic burden. Heart rate variability (HRV) has been studied as a biomarker linking autonomic nervous system (ANS) regulation and cardiovascular health. However, its role remains under investigation, as associations are not always consistent and may attenuate when adjusted for conventional risk factors.

**Figure 1:**
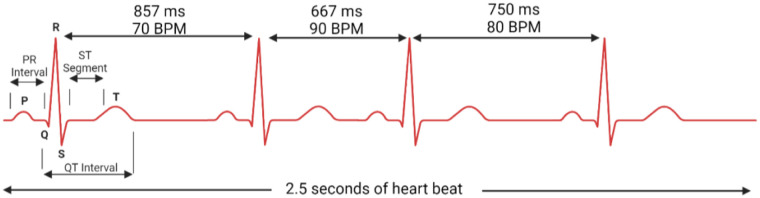
Heart rate variability visualised with R-R interval changes.

HRV is defined as the temporal variation between consecutive heartbeats, often measured as R-R intervals on an electrocardiogram (ECG) (Figure 1) (2). One key component of HRV is respiratory sinus arrhythmia (RSA)—whereby heart rate increases during inspiration and decreases during expiration. RSA is most prominent in young, healthy individuals, reflecting robust autonomic regulation. RSA optimises gas exchange, enhances cardiac efficiency, and stabilises blood pressure (3–5). However, in CVD, RSA is often diminished or lost, indicating autonomic dysfunction (6).

The aim of this review is to provide a structure and balanced synthesis of the role of HRV in cardiovascular disease. We review HRV domains and their clinical applications, evaluate evidence across disease context, and highlight challenges, inconsistencies, and future directions.

## 2 Methods

We constructed a structured literature search to inform this narrative review. Searches were performed in Pubmed databases between January 1980 and December 2024, supplemented by manual screening of reference lists and relevant review articles.

Search strategy: We used combinations of the terms “heart rate variability” OR “HRV” AND “cardiovascular disease,” “myocardial infarction,” “heart failure,” “arrhythmia,” “sudden cardiac death,” “autonomic dysfunction,” in the title and abstract fields.

We included studies published in English, assessing HRV (time-, frequency-, or nonlinear-domain measures) in the context of cardiovascular disease, risk stratification, or treatment outcomes. We include observational studies, randomised controlled trials, and meta-analyses. We excluded case reports, conference abstracts, and animal studies unless directly relevant to mechanistic insights. We also excluded studies without clear HRV methodology.

When assessing the evidence, we prioritised large prospective studies, meta-analyses, and studies with 24-hour Holter recordings. Where only short-term (e.g., 5-minute) HRV data were available, this is explicitly noted. This structured approach aimed to minimise selection bias while acknowledging that the review is narrative rather than systematic.

## 3 HRV metrics and analytical domains

HRV can be assessed using three principal approaches: time-domain, frequency-domain, and non-linear methods, each capturing different aspects of autonomic regulation. In this review, “decreased HRV” refers to values below those in age- and sex-matched healthy control populations, or relative declines within individuals over time. Reduced HRV generally reflects diminished vagal modulation. However, some indices can paradoxically increase in settings of sympathetic overactivation or pathological autonomic instability.

### 3.1 Time-domain measures

Time-domain indices of HRV quantify the variability in the interbeat interval between successive heartbeats (NN intervals) (Table 1). The most widely used include the standard deviation of NN intervals (SDNN), the root mean square of successive differences (RMSSD), and the proportion of adjacent NN intervals differing by >50 ms (pNN50). SDNN is often considered a marker of both sympathetic and parasympathetic influences. RMSSD and pNN50 primarily reflect short-term variability mediated by vagal activity. SDNN from 24-hour Holter recordings consistently predicts mortality after myocardial infarction and in heart failure (7–10).

**Table 1:** Heart rate variability time-domain measures.

Parameter	Unit	Description	Change with decreased HRV	Clinical significance
HR max—HR min	bpm	Average difference between the highest and lowest heart rates during each respiratory cycle.	↓	Reflects overall heart rate adaptability; reduced values indicate impaired autonomic regulation
pNN50	%	Percentage of successive RR intervals that differ by more than 50 ms.	↓	Indicates parasympathetic activity; lower values suggest reduced vagal tone
SDANN	ms	Standard deviation of the average NN intervals for each 5 min segment of a 24 h HRV recording	↓	Reflects long-term variability; reduced values indicate impaired circadian rhythm
SDNN	ms	Standard deviation of NN intervals. Reflects overall variability.	↓	A global measure of HRV; lower values are associated with increased cardiovascular risk
SDNN index (SDNNI)	ms	Mean of the standard deviation of all the NN intervals for each 5 min segment of a 24 h HRV recording	↓	Similar to SDNN but provides a more granular view of variability over time
SDRR	ms	Standard deviation of RR intervals	↓	Reflects overall variability; reduced values indicate impaired autonomic regulation
RMSSD	ms	Root mean square of successive RR interval differences. Indicates short-term variability and parasympathetic activity.	↓	A key marker of vagal tone; lower values suggest reduced parasympathetic values

Bpm, beats per minute; HRV, heart rate variability; Interbeat interval, time interval between successive heartbeats; NN intervals, interbeat intervals from which artifacts have been removed; RR intervals, interbeat intervals between all successive heartbeats.

### 3.2 Frequency-domain measures

Frequency-domain measurements decompose HRV into spectral components (Table 2). The high-frequency (HF) band (0.15–4 Hz) reflects parasympathetic modulation and is closely related to respiratory sinus arrhythmia (11). The low-frequency (LF) band (0.04–0.15 Hz) is considered a marker of both sympathetic and parasympathetic input, although its interpretation remans debated (12). The LF/HF ratio has often been used as a proxy for sympathovagal balance, but this view is controversial, as LF is not a pure marker of sympathetic tone (13–16). Very-low-frequency (VLF) and ultra-low-frequency (ULF) bands require long-term recordings and are sensitive to methodological differences, limiting their reproducibility across studies.

**Table 2:** Heart rate variability frequency-domain measures and changes typical of decreased heart rate variability.

Parameter	Unit	Description	Change with decreased HRV	Clinical significance
ULF power	ms^2^	Absolute power of the ultra-low-frequency band (<0.003 Hz)	↓	Reflects very slow oscillations, often linked to thermoregulation and circadian rhythms
VLF power	ms^2^	Absolute power of the very-low-frequency band (0.0033–0.04 Hz)	↑	Associated with sympathetic activity and hormonal regulation; elevated in stress or disease
LF peak	Hz	Peak frequency of the low-frequency band (0.04–0.15 Hz)	No change/slight shift to higher frequency	Reflects baroreceptor activity; shifts may indicate autonomic imbalance
LF power	ms^2^	Absolute power of the low-frequency band (0.04–0.15 Hz). Reflects both sympathetic and parasympathetic influences.	↑	Indicates sympathetic dominance when elevated; often associated with stress or heart failure
HF peak	Hz	Peak frequency of the high-frequency band (0.15–0.4 Hz). Reflects parasympathetic activity.	No change	Linked to respiratory sinus arrhythmia; stable values suggest preserved vagal tone
HF power	ms^2^	Absolute power of the high-frequency band (0.15–0.4 Hz)	↓	A direct measure of parasympathetic activity; reduced values indicate vagal withdrawal
LF/HF	%	Ratio of LF-to-HF power. Often used as an index of sympathovagal balance.	↑	Higher values indicate sympathetic dominance; associated with stress, anxiety, or CVD

CVD, cardiovascular disease; HF, high frequency; HRV, heart rate variability; LF, low frequency; ULF, Ultra-low frequency; VLF, very low frequency.

The length of ECG data acquisition influences the reliability of HRV indices. Frequency-domain analysis requires sufficient window sizes: while 5-minute recordings allow reliable estimation of high- and low-frequency power, they cannot resolve VLF or ULF components, which generally require 24-hour Holter monitoring. Similarly, time-domain measures such as SDNN are highly dependent on recording duration. Despite suggestions that as little as 10 s of data may provide HRV estimates, this is not recommended for prognostic use (17, 18). The lack of consensus on the minimum recording duration required for reliable HRV analysis further contributes to heterogeneity across studies (2, 13, 19, 20).

### 3.3 Non-linear HRV measures

In addition to time- and frequency-domain approaches, non-linear methods have been developed to capture the complex, fractal-like behaviour of heart rate dynamics (21). These techniques are based on the recognition that cardiovascular regulation is not strictly linear, and that traditional indices may overlook subtle patterns of autonomic control.

One of the most common non-linear approaches is the Poincare plot, a scatterplot of each R-R interval against the subsequent time interval (22). From this, two descriptors are derived: SD1, reflecting short-term variability, and SD2, reflecting long-term variability. A reduction in both SD1 and SD2 have been linked to increased cardiovascular mortality (23).

Detrended fluctuation analysis (DFA) is another non-linear approach, which characterises fractal scaling; lower short-term scaling exponent α1 has been associated with sudden cardiac death (SCD) and heart failure mortality (24, 25). Entropy-based measures, such as multiscale entropy, assess signal complexity; lower entropy typically indicates reduced physiological adaptability and has been associated with worse outcomes in patients with heart failure (26).

Together, time, frequency and nonlinear measures form a complementary toolkit. Understanding their distinctions is essential for critically appraising the HRV literature and interpreting results across cardiovascular disease contexts.

## 4 Physiological development of HRV

**Figure 2:**
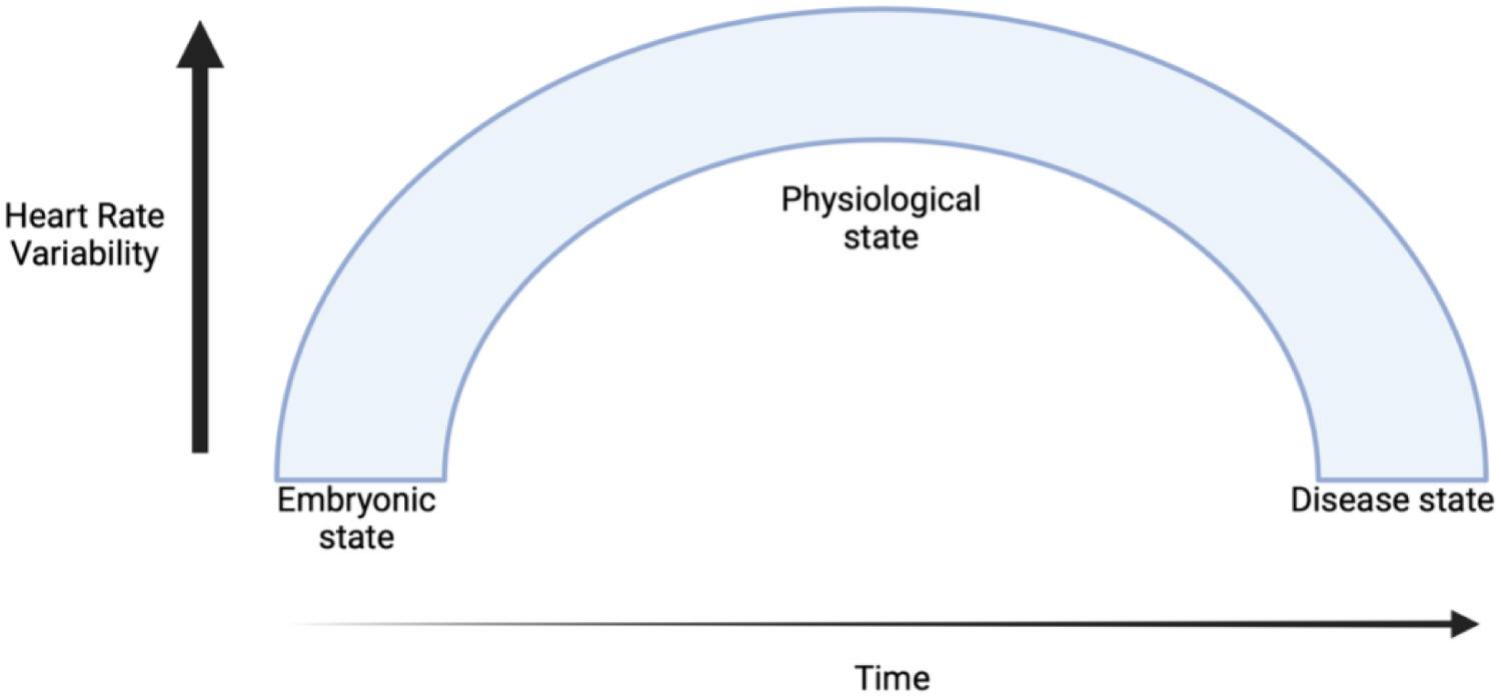
Progression of heart rate variability from embryogenesis through healthy development and into disease states. HRV increases during healthy development, peaking in early adulthood, but declines in cardiovascular disease, reflecting autonomic dysfunction and increased cardiovascular risk.

HRV reflects the dynamic balance between the sympathetic and parasympathetic branches of the ANS, which govern the body's cardiovascular responses to internal and external stimuli. The emergence of HRV begins during embryogenesis, when the foetal heart and ANS gradually mature, and reflects early autonomic regulation of cardiovascular function (27). Throughout healthy development, HRV increases, reaching its peak in early adulthood (Figure 2). A healthy ANS demonstrates high variability in R-R intervals. However, in the presence of heart failure, HRV declines, reflecting autonomic dysfunction and predicts increased risk of SCD (28).

### 4.1 Clinical thresholds, interpretation and application

Clinical thresholds have been proposed to identify patients at elevated cardiovascular risk. The European Society of Cardiology suggest that SDNN <50 ms represent severely depressed HRV, with <100 ms considered moderately reduced (19). For short-term recordings, RMSSD <15 ms suggests parasympathetic deficiency. However, thresholds vary by age, sex, and recording conditions (29). Importantly, abnormally elevated HRV may represent dysregulated rather than optimised autonomic function. Some studies have observed U-shaped relationships between HRV and outcomes, particularly in elderly populations (30–33).

**Figure 3:**
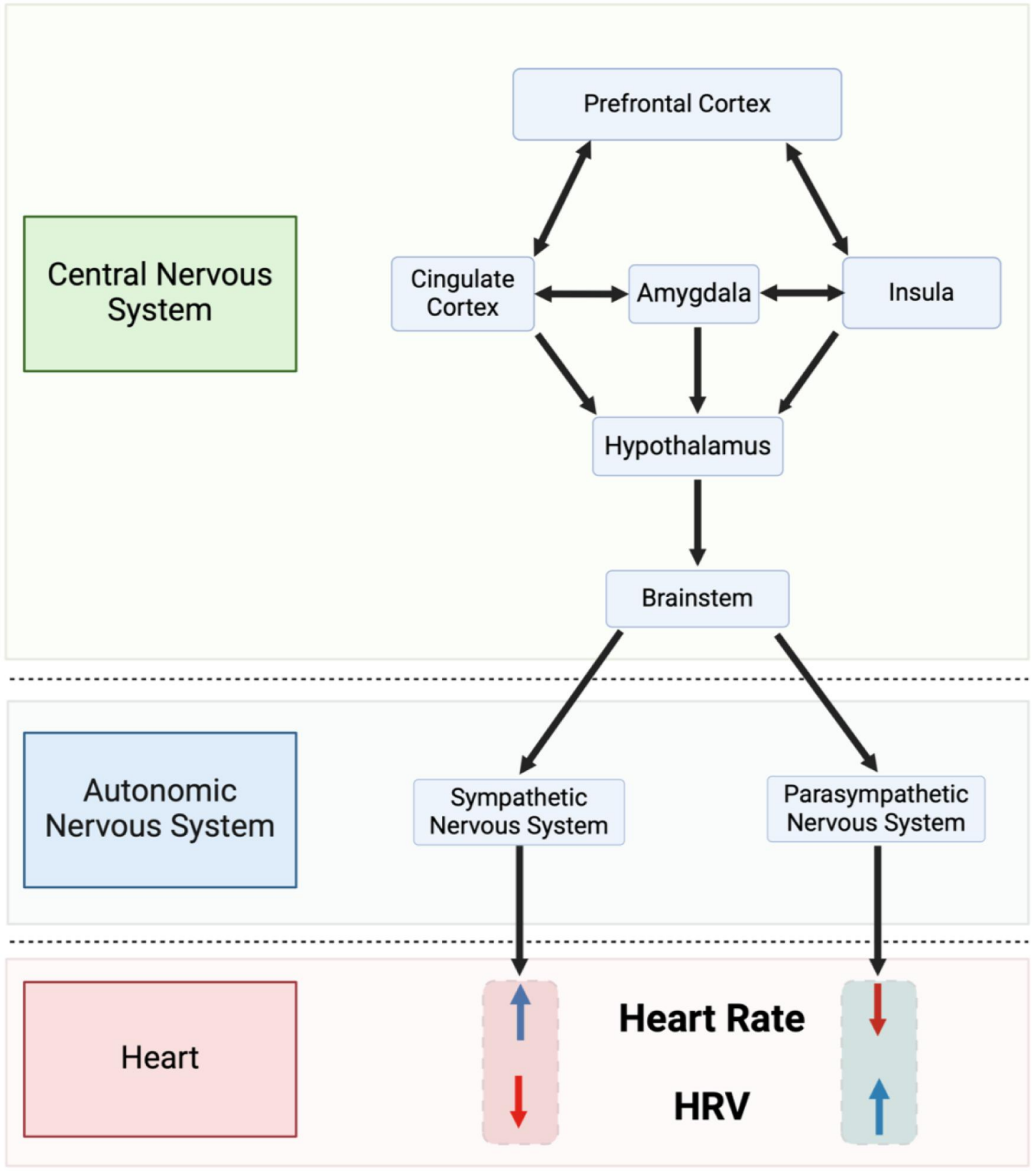
Simplified depiction of the neurovisceral integration model described by thayer and sternberg (35). HRV, heart rate variability.

The significance of HRV lies in its ability to serve as a sensitive and early indicator of autonomic dysfunction, often preceding overt clinical manifestations of disease (34). Unlike static measures such as blood pressure or cholesterol levels, HRV captures real-time fluctuations in autonomic activity (Figure 3).

## 5 HRV as a diagnostic marker

HRV provides an accessible, non-invasive method to quantify autonomic imbalance, but interpretation assumes intact sinus node function and stable atrioventricular (AV) conduction. In conditions such as sick sinus syndrome, atrial fibrillation (AF), or advanced AV block, variability in R-R intervals no longer reflects autonomic modulation or sinus rhythm. Instead, irregularity may arise from intrinsic nodal dysfunction or arrhythmic events.

### 5.1 Metabolic dysregulation

Reduced HRV has been associated with metabolic abnormalities. The Toon Health Study (2009–2012) assessed 1,899 individuals without diabetes medication using a 75-gram oral glucose tolerance test and short-term (5-minute) HRV recordings (36). Lower HRV metrics correlated with higher indices of insulin resistance. However, HRV explained only a small proportion of the variance in metabolic outcomes. The study also has broader limitations. The cross-sectional design prevents establishing causality, and the study's homogenous Japanese population limit generalisability. Future longitudinal studies with diverse populations would strengthen these findings and clarify whether HRV changes precede or follow metabolic dysfunction.

### 5.2 Inflammation

Reduced HRV has also been associated with heightened localised and systemic inflammation, which are recognised contributors to atherosclerosis and other cardiovascular complications (37, 38). First described by Tracey in 2002, the “inflammatory reflex” is a complex of efferent signals in the vagus nerve suppressing macrophage-mediated release of peripheral cytokines in response to inflammatory triggers (39). A meta-analysis by Williams et al. (2019) clarified the relationship between HRV and inflammation, identifying that higher HRV, particularly vagal HRV, was associated with lower levels of inflammation. A maladaptive diminishing of parasympathetic nervous system activity and overactivity of the sympathetic nervous system has been proposed to contribute to a pro-inflammatory state (40).

### 5.3 Psychological stress and depression

Chronic stress, which disrupts ANS balance, has been linked to both reduced HRV and an increased incidence of CVD (41). A meta-analysis by Kim et al. (2018) surveyed studies that provided a rationale for using HRV as a psychological stress indicator and identified that the most frequently reported factor contributing to changes in HRV variables was a reduction in parasympathetic activity (41). Rebalancing the ANS through vagal stimulation has been shown to limit infarct size and inflammatory response to myocardial ischaemia and reperfusion in male rats that underwent myocardial ischaemia for 30 min and reperfusion for 24 h (42). The anti-inflammatory and anti-apoptotic properties of the nicotinic pathway were proposed to be the underlying mechanism.

### 5.4 Electrophysiological disorders

HRV has been explored as a marker of arrhythmic risk. A meta-analysis identified that patients with low HRV without known CVD have a 32%–45% increased risk of a first cardiovascular event, although heterogeneity was substantial and incremental predictive value was unclear (19). Most evidence derives from older cohorts, and relevance in the era of reperfusion and guideline-directed therapy is uncertain.

### 5.5 Coronary artery disease

Patients with documented CAD exhibit reduced HRV, reflecting altered autonomic regulation of cardiac function. This reduction appears across multiple HRV indices, including time-domain measures and frequency-domain parameters (43, 44). In the ARM-CAD study, there was a linear relationship between CAD severity and LF power, regardless of anatomical location of coronary stenoses (44). Similar findings were reported in Feng et al. (45). Unlike the ARM-CAD study, Feng and colleagues identified that the degree of reduction in time-domain HRVs was dependent on CAD location. The studies did demonstrate that HRV might serve as a non-invasive indicator of disease burden. However, limitations include small sample sizes and short-term recordings, which may limit generalisability.

## 6 HRV as a prognostic tool

HRV has been studied as a prognostic tool in cardiovascular disease. Reduced variability across time- and frequency-domain indices has been associated with higher risk of mortality, arrhythmias, and recurrent cardiac events. However, findings are inconsistent, and predictive value often diminishes after accounting for conventional risk factors.

### 6.1 Predicting adverse outcomes

Lower HRV indices have been associated with increased risk stroke, SCD and mortality after an MI (46–48). In patients with diabetes or hypertension, reduced HRV is associated with an increased likelihood of silent myocardial ischaemia (49). In heart failure populations, depressed HRV reflects sympathetic activation and vagal withdrawal and is consistent with arrhythmic death (50). Nonetheless, HRV has not consistently improved risk prediction models beyond established variables. Methodological heterogeneity—retrospective analyses, variable follow-up, and differing HRV protocols—limits comparability.

### 6.2 Temporal patterns

Beyond average values, emerging evidence suggests that temporal instability in HRV may add prognostic information. In post-MI cohorts, decreased day-to-day stability predicted mortality independent of mean HRV values (51). Similarly, in heart failure, abrupt declines in HRV preceded acute decompensation by several days, suggesting a role for continuous monitoring (52).

### 6.3 Specific cardiovascular conditions

#### 6.3.1 Post-MI

A study by Zuanetti and colleagues (1996) identified that in patients who received thrombolysis following an MI, reduced values for all time-domain indexes of HRV were predictive for higher risk of mortality (53). More recently, a prospective cohort study by Pukkila et al., (2025) evaluated repeated 24-hour HRV recordings in post-MI patients and found that HRV parameters correlated with infarct severity and left ventricular function, but their independent prognostic value for future cardiac events diminished after adjustment for left ventricular ejection fraction and GRACE score (54). These findings suggest that HRV may complement, rather than replace, established post-MI risk-stratification tools. Supporting this, Karp and colleagues’ (2009) showed that even ultra-short measurements (10 s ECG segments obtained at admission) predicted two-year mortality after MI (55), indicating that brief HRV assessments can still provide meaningful prognostic information.

#### 6.3.2 CAD

HRV has shown prognostic value in patients with CAD. Tsuji and colleagues (1996) analysed ECG recordings from Framingham Heart Study patients who were free of clinically apparent CAD or congestive heart failure to assess the relationship between HRV metrics and the risk of heart disease (56). Lower HRV was associated with an increased risk of a new cardiac event, demonstrating that HRV by ambulatory monitoring offers prognostic information beyond that provided by the evaluation of traditional CVD risk factors.

The prognostic potential of HRV extends to patients undergoing revascularisation procedures. Thanh et al. (2023) investigated the role of pre-operative HRV in predicting AF in patients undergoing coronary artery bypass grafting (57). They found that reduced pre-operative HRV—particularly lower time-domain measures such as SDNN and RMSSD—predicted post-operative AF within 7 days of surgery, but these associations diminished over longer follow-up periods. Frequency-domain components such as HF power, were not predictive. These findings suggest that impaired overall autonomic variability, rather than isolated parasympathetic withdrawal, contributes to post-operative AF risk in the immediate recovery phase.

#### 6.3.3 Ventricular arrhythmias and SCD

Reduced HRV predicts ventricular arrhythmias and SCD in high-risk cohorts. In a prospective study by Galinier and colleagues, 190 patients with chronic heart failure in sinus rhythm were followed for 22 months (58). The study found that a daytime LF power of < 33 ln(ms2) was an independent predictor of SCD, with a relative risk of 2.8. For AF, associations are more complex. Contrary to the linear relationship observed with ventricular arrhythmias, the Multi-Ethnic Study of Atherosclerosis (MESA) study found that both reduced and excessive HRV parameters increased AF risk, with the highest incidence in individuals at either extreme (59). This finding highlights a broader issue: HRV relationships with outcomes are sometimes U-shaped or paradoxical, complicating interpretation and limiting its reliability as a standalone biomarker.

#### 6.3.4 Congestive heart failure

La Rovere and colleagues (2003) identified that short-term HRV strongly predicted SCD in heart failure patients (28). Specifically, they found that reduced LF power of HRV was associated with an increased risk of SCD, independent of other established risk factors. Beyond SCD, persistently low HRV in heart failure patients is also associated with recurrent hospitalisation (60). This association indicates that deteriorating autonomic function may precede clinical decompensation, potentially providing an opportunity for pre-emptive intervention.

## 7 Therapeutic contexts

HRV has been explored not only as a biomarker but as a target for therapeutic modulation. Evidence is promising across lifestyle, pharmacological, and device-based interventions, yet findings are inconsistent and clinical translation remains limited.

### 7.1 Behavioural and lifestyle interventions

Exercise-based interventions have demonstrated benefits in improving HRV. Regular aerobic exercise, such as walking, cycling, or swimming, has been shown to enhance HRV (61). HIIT has been identified as a potential strategy to enhance HRV in post-MI patients. HIIT has been associated with modest improvements in HRV metrics, although the clinical relevance of these findings remains uncertain (62).

Dietary modifications have been associated with differences in autonomic function. In a twin study of 276 men, adherence to a Mediterranean diet was linked with higher HRV across multiple time- and frequency-domain indices, suggesting improved autonomic balance and potentially lower cardiovascular risk (63). Smoking has been associated with blunted vagal modulation and higher sympathetic dominance, reflected by lower SDNN and RMSSD and higher LF/HF ratios in long-term smokers (64). These studies highlight associations between lifestyle factors and HRV; however, they are observational, and causality cannot be inferred.

By providing real-time feedback on autonomic status, HRV monitoring—particularly when integrated into wearable technologies—may help tailor personalised exercise, stress-management and recovery strategies (65, 66). However, it remains unclear whether changes in HRV actively mediate the clinical benefits of these lifestyle interventions ore merely reflect parallel physiological adaptations such as improved metabolic function, reduced inflammation, or enhanced cardiovascular fitness.

### 7.2 Autonomic modulation therapies

Recent advances in biofeedback therapy, pharmacological agents, and VNS have highlighted the potential benefits of HRV modulation in clinical practice.

#### 7.2.1 Biofeedback therapy

**Figure 4:**
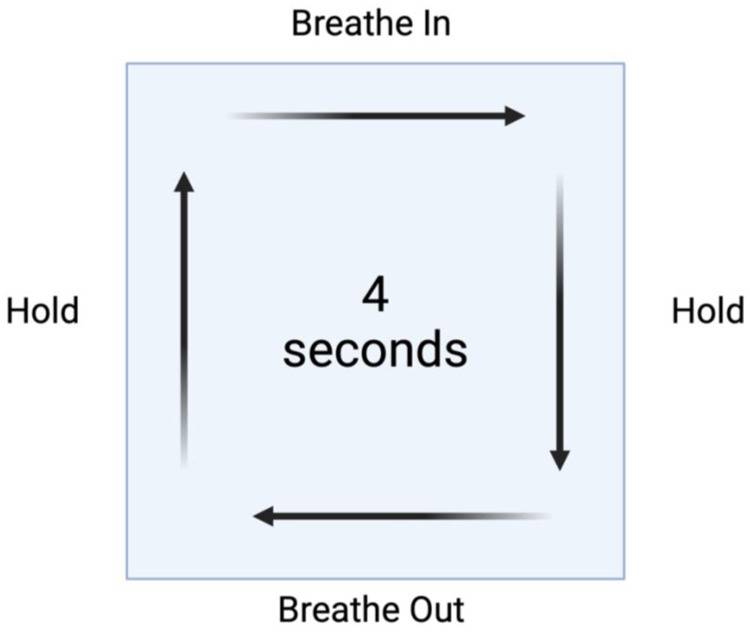
Box breathing method for heart rate variability. This method involves inhaling, holding the breath, exhaling, and holding again for equal durations, typically following a 4-4-4-4 s pattern.

Biofeedback therapy employs real-time physiological feedback to enhance autonomic regulation. HRV biofeedback focuses on synchronising breathing patterns with heart rate to increase parasympathetic activity (67). A common breathing technique used in HRV biofeedback is box breathing (also known as square breathing), which involves inhaling, holding the breath, exhaling, and holding again for equal durations, typically following a 4-4-4-4-second pattern (Figure 4).

Although direct peer-reviewed evidence for box-breathing remains limited, slow-paced breathing at approximately 5–7 breaths per minute has been shown to increase HRV indices such as RMSSD and HF power, reflecting enhanced parasympathetic tone and vagal modulation of HRV (67, 68). Such breathing-based HRV biofeedback interventions have also been associated with improvements in mood and reductions in perceived stress. Paced-breathing techniques are therefore potential tools within HRV biofeedback to promote autonomic balance and psychological wellbeing.

#### 7.2.2 Pharmacological modulation

Pharmacological agents can play a role in modulating HRV by targeting specific autonomic pathways. Beta-blockers have been shown to improved HRV metrics in heart failure patients (69). Similarly, angiotensin-converting enzyme inhibitors and angiotensin receptor blockers have demonstrated efficacy in improving HRV by modulating neurohormonal activity, particularly in patients with heart failure and hypertension (70, 71). These agents decrease systemic inflammation and oxidative stress, through inhibition of reactive oxygen species, CRP and pro-inflammatory interleukins, indirectly contributing to enhanced autonomic regulation (72, 73).

#### 7.2.3 VNS

VNS is a device-based therapy that activates the vagus nerve to enhance parasympathetic output. Preclinical studies in animal models—including in rats and canines—have shown that both invasive (implantable) and non-invasive (transcutaneous) VNS can improve HRV and attenuate cardiac dysfunction (74, 75). A Japanese study in rabbits found that intermittent VNS, but not constant VNS, increased the vagal component of HRV (76). Translation of these findings into human applications remains challenging. The ANTHEM-HF study (2014) showed promising results in humans with heart failure, but the NECTAR-HF trial demonstrated improvements in quality of life without significant effects on cardiac remodelling, highlighting the uncertainties about translation from animal models (77, 78).

### 7.3 Disease-specific applications

#### 7.3.1 Ventricular arrhythmias and SCD prevention

Pharmacological agents that modulate autonomic tone—particularly beta-blockers—reduce sympathetic activity and improve overall cardiac stability. While these agents have been shown to increase HRV in heart-failure populations, a large meta-analysis of 24,799 patients across 30 randomised control trials attributes the 31% reduction in SCD risk to the drug's direct anti-arrhythmic effect, rather than a proven causal link with HRV improvement (79).

Other autonomic modulation techniques, such as renal denervation and cardiac sympathetic ablation, aim to reduce excessive sympathetic drive that predisposes to arrhythmias (80–82). Evidence of HRV improvement following these procedures is limited, and most data comes from small series or case reports (83, 84). Such interventions illustrate the potential role of therapies that block sympathetic activity in arrhythmia management, though their relationship with HRV changes remain largely theoretical due to limited or absent HRV measurement within these studies.

#### 7.3.2 CAD management

HRV analysis could become an integral component of comprehensive CAD management, informing decisions across the spectrum of care from primary prevention to post-event rehabilitation. In primary prevention, HRV assessment might help identify individuals with subclinical autonomic dysfunction who may benefit from more aggressive risk factor modification. Hillebrand et al. (2013) found that individuals with low HRV had a 32%–45% increased risk of first cardiovascular events, suggesting that HRV could enhance traditional risk stratification models (85). Manresa-Rocamora and colleagues showed that HRV-guided training improves HRV parameters to a greater extent than predefined training in patients with CAD (86). These findings demonstrate that HRV could serve as a valuable surrogate endpoint for assessing the efficacy of rehabilitation interventions.

#### 7.3.3 Congestive heart failure

In heart failure patients, interventions that improve HRV—such as VNS and beta-blockers—have been associated with improved cardiac output (69, 77, 87, 88). These benefits reflect improved baroreflex sensitivity, stabilisation of autonomic control, and enhanced myocardial efficiency. Recent advancements in pacing technology have further highlighted the potential of HRV modulation in heart failure. Shanks et al. (2022) demonstrated the therapeutic benefit of restoring RSA in an ovine model of heart failure with reduced ejection fraction (89). The study found that RSA pacing significantly increased cardiac output by 1.4 L/min (20%) compared to conventional monotonic pacing. This improvement was accompanied by a reduction in apnoeas, reversal to cardiomyocyte hypertrophy, and restoration of T-tubule structure, which are critical for force generation in cardiomyocytes. Importantly, the study also observed a decrease in systemic vascular resistance, suggesting that RSA pacing not only enhanced cardiac function but also promotes reverse remodelling.

### 7.4 Mental health interventions

Depression, anxiety, and chronic stress are associated with autonomic dysregulation and increased CVD risk. HRV serves as a measurable link between psychological and cardiovascular health, offering insights into how mental health interventions influence autonomic balance. A meta-analysis by Pizzoli and colleagues (2021) identified that HRV biofeedback in depressed patients increased HRV levels, closer to that observed in healthy populations, and there was a concomitant improvement in depressive symptoms (90). Biofeedback in combination with usual treatment led to a greater reduction in depression symptoms compared to the group who received only regular treatment. In addition, the group that received biofeedback also showed increased HF HRV during both resting and stress conditions, indicating improved ANS function (91).

Other mental health interventions, such as mindfulness-based stress reduction (MBSR) and cognitive-behavioural therapy, have also demonstrated efficacy in improving HRV in patients with CVD (92, 93). It is thought that MBSR techniques, which involve meditation and focused breathing, enhance parasympathetic activity and reduce sympathetic overdrive (94). Cognitive-behavioural therapy, targets negative thought patterns and behaviours that contribute to stress, driving significant HRV improvements following therapy (92).

## 8 Challenges and risks associated with HRV modulation

### 8.1 Patient heterogeneity

The efficacy of interventions like biofeedback or VNS is influenced by baseline autonomic function, age, comorbidities, and psychological health play. Patients with severely impaired autonomic systems or those with chronic conditions like diabetes may not respond as effectively as healthier individuals. Psychological factors such as stress, anxiety or depression can confound HRV outcomes, complicating the interpretation of results and necessitating personalised approaches.

### 8.2 Methodological variability

The absence of standardised protocols for HRV measurement and intervention adds to the complexity of its application. Inconsistencies in HRV findings across studies often stem from methodological heterogeneity, including differences in data acquisition duration (short-term vs. 24-hour recordings), and in the analytical domain applied (time-, frequency-, or non-linear measures) (2, 13, 66). Furthermore, disparities in the performance of wearable HRV monitors vs. traditional ECG systems contribute to inconsistent reliability, creating barriers for widespread adoption (65, 66). Standardising these protocols is essential for ensuring consistency, reliability, and comparability.

Accurate HRV assessment relies on precise detection of R waves and correct identification of R-R intervals. Signal noise, reduced R-wave amplitude, and ectopic activities such as premature atrial or ventricular contraction can distort variability metrics. Although most analysis software incorporates artefact detection and correction algorithms, these approaches differ in stringency, and residual errors can bias HRV outcomes, particularly in patients with arrhythmias or conduction abnormalities. Transparent reporting of preprocessing steps is therefore essential for reproducibility.

### 8.3 Limited long-term data

The short-term focus of most studies on HRV modulation presents another limitation. While immediate challenges in HRV indices have been observed following interventions such as biofeedback or VNS, the long-term benefits of these changes remain uncertain. Questions persist regarding the sustainability of improved HRV over months or years, as well as the durability of associated clinical outcomes like reduced hospitalisations or improved quality of life.

Similarly, the long-term safety of some interventions, especially invasive techniques like VNS, remains insufficiently studied. Risks such as nerve damage, infections, or unintended effects on other autonomic functions warrant thorough investigation through longitudinal research. However, non-invasive techniques such as box-breathing are already being integrated into the programming of wearable technology and present a much lower-risk method of integrating HRV modulation techniques into everyday life.

### 8.4 Potential adverse effects

Potential adverse effects pose risks, even for non-invasive HRV modulation techniques. Although biofeedback therapy is generally safe, some patient(s may experience frustration or dependency when desired HRV improvements are not achieved (95). VNS, whether invasive or transcutaneous, can result in side effects such as dizziness, hoarseness, and, in rare cases, complications from the implantation process (77, 78). Pharmacological interventions that enhance HRV, such as beta-blockers, can cause side effects such as bradycardia, fatigue, or mood disturbances. A careful risk-benefit analysis is therefore essential.

### 8.5 Ethical and accessibility concerns

Ethical and accessibility concerns also emerge with the increasing reliance on wearable devices and advanced technologies. The high cost of devices and therapy sessions may limit access for underserved populations, exacerbating healthcare disparities. Data privacy is another concern, as wearable HRV monitoring generates large volumes of sensitive health information that could be vulnerable to breaches. Moreover, over-reliance on automated systems for HRV analysis without adequate clinical oversight could result in suboptimal or inappropriate treatment strategies. Equitable healthcare policies, robust cybersecurity measures, and integrated clinical-patient decision-making frameworks are needed to address these issues.

### 8.6 Technological challenges

Technological challenges further complicate the implementation of HRV modulation. While wearable devices offer convenience, their accuracy in capturing subtle HRV changes often falls short of traditional ECG systems. Machine learning algorithms, which play a significant role in analysing HRV data, may suffer from biases if trained on non-representative datasets. Additionally, achieving real-time therapeutic adjustments based on HRV monitoring is technologically demanding, requiring advanced processing capabilities and seamless device interoperability.

#### 8.6.1 Translational gaps between research and clinical practice

Finally, there is a translational gap between research and clinical practice. Despite evidence supporting HRV modulation, its integration into routine healthcare remains limited. Many clinicians are unfamiliar with HRV metrics and their clinical significance, while resource constraints in healthcare systems hinder the adoption of new technologies. Bridging this gap will require educational initiatives to train clinicians, improved user-friendly devices, and scalable implementation strategies that can be adapted across diverse healthcare settings.

## 9 Future directions

The exploration of HRV in CVD management has uncovered numerous applications and opportunities. However, several areas warrant further investigation to maximise its potential. These future directions include developing personalised therapeutic protocols, integrating artificial intelligence (AI) and big data, and expanding applications to comorbid conditions.

### 9.1 Personalised therapeutic protocols

Personalised HRV modulation strategies could integrate genetic, lifestyle, and physiological data to create individualised care plans. Genomic studies could identify polymorphisms influencing autonomic regulation and HRV responsiveness. Combining lifestyle modifications with continuous monitoring may optimise therapeutic outcomes. Furthermore, individual variations in baseline autonomic function and disease progression necessitate adaptive protocols. Tailored biofeedback therapies, pharmacological regimens, or device-based interventions could enhance efficacy while minimising side effects.

For patients with ICDs, there is emerging interest in exploring how HRV metrics might potentially inform device programming strategies. The autonomic information provided by HRV analysis could potentially complement traditional programming approaches, with the goal of reducing inappropriate shocks while maintaining protective efficacy against life-threatening arrhythmias, as well as optimising the individual's quality of life. This area remains largely theoretical and requires robust prospective studies before clinical application but represents a promising direction for research that could ultimately improve patient quality of life without compromising patient safety.

### 9.2 Integration of AI and big data

Another promising avenue is the integration of AI and big data analytics, which are poised to greatly advance HRV-based interventions. AI can process complex HRV metrics, uncovering patterns that predict disease progression or treatment response. Real-time algorithms could dynamically adjust therapeutic interventions based on HRV trends. By integrating HRV with other biomarkers, predictive models could stratify patients by risk and recommend proactive measures.

### 9.3 Comorbid conditions

HRV modulation also holds promise beyond primary cardiovascular disorders, particularly in managing conditions where autonomic dysfunction is a key component. Future research should explore its role in diabetes management, where HRV-based interventions could mitigate the progression of diabetic neuropathy and improve glucose regulation. In mental health disorders, HRV-guided therapies, including biofeedback and VNS, may enhance outcomes. The anti-inflammatory effects of enhanced parasympathetic activity suggest a role for HRV modulation in conditions like rheumatoid arthritis and fibromyalgia.

### 9.4 Long-term and multi-dimensional studies

Expanding HRV applications necessitates robust, long-term studies to validate efficacy across diverse populations and settings. Studies should also assess the synergistic effects of combining HRV modulation with traditional therapies like pharmacological treatments or physical rehabilitation. Moreover, multi-dimensional outcomes, including psychological well-being, systemic inflammation, and healthcare costs, should be evaluated to comprehensively assess the impact of HRV-based strategies.

### 9.5 Standardisation

The challenges outlined in Section 8 of this manuscript highlight a core barrier to HRV's clinical translation: heterogeneity at every stage of acquisition, analysis and interpretation. These inconsistencies make comparison between studies difficult and limit the reliability of HRV as a diagnostic or prognostic tool. Consequently, standardisation is a critical prerequisite for the clinical adoption of HRV analysis. Before any composite “HRV Score” can be considered, the underlying parameters and acquisition methods must be harmonised. Current inconsistencies in recording duration, sampling frequency, and artefact correction significantly affect reproducibility. A valid framework would require standardised criteria for:

- Data acquisition—minimum sampling frequency (e.g., >250 Hz), specification of ECG channel configuration, and signal preprocessing protocols including noise filtering and artefact removal.
- Analytical consistency—verification of signal stationarity, consistent segment lengths for frequency analysis, and transparent reporting of the algorithms used for spectral decomposition or non-linear metrics.
- Patient stratification—definition of population subgroups by age, sex, comorbidities, and medication use to ensure comparability across studies.

Only after these techniques and clinical standards are implemented can a multi-parametric HRV composite score be developed. Such a composite should rely on validated, reproducible metrics weighted according to independently verified prognostic strength. A consensus-driven framework would help ensure that any proposed HRV index or “score” truly reflects physiological reality.

## 10 Conclusion

The future of HRV in CVD management lies in its integration into personalised, data-driven, and multidisciplinary approaches. By harnessing advances in technology and expanding its applications, HRV may contribute to cardiovascular care as an adjunctive tool. However, its widespread adoption will depend on resolving issues of standardisation, reproducibility, and demonstration of added prognostic value beyond established risk factors.
